# Inflammatory Insights: Analysis of a Fecal Biomarker in Neurodegenerative and Gastrointestinal Disorders

**DOI:** 10.3390/biomedicines13102411

**Published:** 2025-10-01

**Authors:** Anca Chisoi, Nicolae Dobrin, Georgeta-Camelia Cozaru, Anita-Cristina Ionescu, Mariana Aschie, Lidia Kajanto, Sabina Elena Vlad, Manuela Enciu, Ion Alexandru Popovici, Bogdan Cîmpineanu

**Affiliations:** 1 “Sf. Apostol Andrei” Emergency County Hospital, Tomis Bulevard 145, 900591 Constanta, Romania; anca.chisoi@365.univ-ovidius.ro (A.C.);; 2 Center for Research and Development of the Morphological and Genetic Studies of Malignant Pathology (CEDMOG), “Ovidius” University of Constanta, 900591 Constanta, Romania; 3 Academy of Romanian Scientists, 3 Ilfov Street, 050094 Bucharest, Romania; 4 Oncological Institute “Prof. Dr. Alexandru Trestioreanu”, 022328 Bucharest, Romania; 5 Faculty of Medicine, “Ovidius” University of Constanta, 1 Universitatii Street, 900470 Constanta, Romania; 6 Research Center of the Natural Sciences Department, Ovidius University of Constanta, 1 Universitatii Street, 900470 Constanta, Romania; 7 Faculty of Dentistry, Carol Davila University of Medicine and Pharmacy, 17-23 Calea Plevnei, 010221 Bucharest, Romania

**Keywords:** inflammatory bowel disease, Parkinson’s disease, inflammation, calprotectin

## Abstract

**Background/Objectives**: Inflammatory bowel disease (IBD) and Parkinson’s disease (PD) are chronic disorders affecting different organs, and evidence has proposed a bidirectional link between them. Fecal calprotectin, which reflects intestinal inflammation and gut barrier injury, is augmented in different neurodegenerative diseases, including PD. In this study, we aimed to investigate whether fecal calprotectin levels in PD increase independently of gastrointestinal inflammation and aging. **Methods:** Fecal calprotectin values were compared in two groups (PD and IBD) of 30 patients each, taking into consideration variables such as endoscopic aspects and age. Analyses of fecal calprotectin levels in PD patients show an increase with advancing age, which was not observed in the IBD cohort. **Results:** Most PD patients had fecal calprotectin values exceeding 200 μg/g (mean value 366.25 μg/g), and their levels were comparable to those of patients with inflammatory bowel disease (Ulcerative Colitis, Crohn’s Disease, mean value 537.70 μg/g). In the PD cohort, a weak positive association between calprotectin levels and inflammatory signs was observed, and in the IBD cohort, higher calprotectin levels were strongly associated with more severe endoscopic modifications. **Conclusions:** This weak but positive correlation with intestinal inflammation in PD patients suggests the involvement of complex mechanisms, other than those related to inflammaging or gut barrier injuries.

## 1. Introduction

Parkinson’s disease (PD) is a progressive neurodegenerative disorder characterized by the gradual development of both motor and non-motor dysfunctions. The pathological hallmark of PD is the presence of Lewy bodies within dopaminergic neurons of the substantia nigra in the midbrain, which leads to their progressive degeneration. The loss of dopaminergic neurons in PD primarily occurs in the substantia nigra pars compacta (SNc) with projections to the striatum [1]. Among neurodegenerative disorders, PD is one of the most prevalent, affecting about 1% of individuals over the age of 65 [2,3].

Parkinson’s disease is characterized by alpha-synuclein misfolding with subsequent intraneuronal amyloid formation and accumulation, low-grade neuroinflammatory changes, and selective neurodegeneration. Available evidence suggests that the pathology usually begins with the gut and olfactory mucosa and spreads to the brain via the vagus and olfactory nerves [4].

Most PD is developed between the ages of 60 and 65, though juvenile PD is designated at less than 21 years of age [5,6]. Old age is a key risk factor for the development and progression of PD, influencing both its onset and severity [7].

Non-motor symptoms such as constipation and dysfunction of gastrointestinal tract (GIT) motility, together with medications used in the management of PD, affect gut microbiota. Alterations in the gut microbiota leading to dysbiosis can cause significant disruption of the gut barrier, which, in turn, triggers systemic inflammation and subsequent neuroinflammation [8]. Neuroinflammation contributes to the pathogenesis of PD through impairment of the blood–brain barrier. Several biomarkers are employed in the diagnosis and prognosis of PD. Among them, calprotectin, a marker of intestinal inflammation and gut barrier damage, is elevated in various neurodegenerative diseases, including PD [8,9,10]. Calprotectin was first identified in 1980 as an antimicrobial protein acting via zinc sequestration, but it is now recognized as both antibacterial and antifungal through its ability to sequester iron and manganese. Representing nearly 60% of neutrophil cytosolic proteins, calprotectin plays a central role in inflammatory responses [11]. Calprotectin is detected in both the blood and feces, but fecal calprotectin is more commonly measured and is regarded as a biomarker of inflammatory bowel disease (IBD). The increase in fecal calprotectin reflects intestinal inflammation, as it originates from leukocytes shed into the gut lumen [8]. Since fecal calprotectin remains at a normal level in irritable bowel syndrome (IBS), this biomarker can be used to distinguish between IBS and IBD [12]. Nonetheless, fecal calprotectin elevation does not distinguish between IBD subtypes and may also be elevated in colorectal cancer [13]. Thus, although not highly specific for IBD, fecal calprotectin serves as a useful screening marker for intestinal inflammation and a prognostic tool for assessing mucosal healing [8]. Recent studies have demonstrated increased fecal calprotectin in patients with PD, indicating the presence of gut inflammation and barrier dysfunction [14]. Both IBD and PD are chronic, progressive disorders affecting distinct organ systems, yet growing evidence supports a bidirectional relationship between gastrointestinal inflammation and PD through the gut–brain axis [15].

In this study, we aim to investigate whether fecal calprotectin levels in patients with Parkinson’s disease increase independently of gastrointestinal inflammation associated with disease and aging. Furthermore, we seek to determine whether these elevations can serve as a potential diagnostic biomarker for Parkinson’s disease.

Since calprotectin is primarily a marker of gastrointestinal damage, we selected a cohort of patients with a diagnosis of IBD and a cohort of patients with a diagnosis of PD. Both conditions are known to involve the gut–brain axis. We aimed to examine the differences in calprotectin levels between these groups and explore the potential role of fecal calprotectin in PD.

## 2. Materials and Methods

### 2.1. Participants, Data Collection, and Ethical Statement

Participants were recruited from the departments of Clinical County Hospital “St Apostle Andrew” Constanta, Romania, and fecal calprotectin values were measured in the CEDMOG research center of “Ovidius” University of Constanta. Study enrolment was based on predefined inclusion and exclusion criteria. Written informed consent, compliant with the Declaration of Helsinki, was obtained from all participants before enrolment, and the study protocol was approved by the hospital Ethics Committee (decision no. 27/29 December 2023). All personal identifiers were removed from samples to ensure patient confidentiality.

The study compared fecal calprotectin values between patients with IBD and those with PD. A healthy control group was not included, as the primary objective was to analyze differences between the two pathologies, and reference values in healthy populations are already well known in the literature.

Inclusion criteria in the IBD group were a clear diagnostic (colonoscopy’s pathognomonic aspect and fecal calprotectin level) of UC or CD. Exclusion criteria for the IBD group were the presence of clinical motor or nonmotor markers for prodromal PD [16] or other chronic neurological diseases, antibiotic treatment or use of other drugs or supplements that may interfere with the results of the testing (steroidal or non-steroidal anti-inflammatory drugs, proton pump inhibitors—PPI) within the past 3 months or recent (less than 6 months prior to study) systemic conditions. On the other hand, to be included in the PD group, patients had to fulfill the Movement Disorder Society (MDS) Clinical Diagnostic Criteria. The following exclusion criteria were applied: recent (less than 6 months prior to study) or concurrent gastrointestinal or systemic conditions, and antibiotic treatment or use of other drugs or supplements that may interfere with the results of the testing (steroidal or non-steroidal anti-inflammatory drugs, PPI) within the past 3 months. A total of 7 patients were excluded (3 from the IBD group—treatment with PPI or antibiotics 3 months prior to the study, and 4 from the PD group—gastrointestinal symptoms or antibiotic treatment 3 months prior to the study). The clinical evaluation was performed by a gastroenterologist for the IBD patients’ group and by a neurologist for the PD patients’ group and included medical history, full neurological examination, and assessment of Parkinsonism using the Unified Parkinson’s Disease Rating Scale (UPDRS) part III [17]. All other relevant data were obtained from medical records for both PD and IBD groups.

### 2.2. Specimen Collection and Analytic Method Used for Fecal Calprotectin Detection

Participants collected their own samples from spontaneously emitted stools in a sterile leak-proof plastic container without a carrier medium, provided by the research team. All patients were provided with written instructions for proper sample collection, storage, and transportation to ensure consistency and minimize preanalytical variability. Immediately after collection, samples were stored by the participants at refrigerating temperature (4–8 °C for a maximum of 12 h) and delivered to the laboratory within one hour, at ambient temperature. In the laboratory, samples provided by patients were labeled, kept at ambient temperature (20–24 °C), and analyzed on the same day (maximum 5 h). Prior to the analysis, stool sample extraction was performed using a 980 IU extraction buffer along with 20 mg of stool, stirred for 30 s, and then centrifuged at 2000× *g* for 10 min using the Thermo Scientific SL16R centrifuge (Thermo Scientific, Waltham, MA, USA). The dilution of the samples was then performed with 980 µL of sample buffer and 20 µL of extraction supernatant. The exact 20 mg of stool was weighed using a laboratory balance (Kern EMS 300-3 (Kern & Sohn GmbH, Balingen, Germany) laboratory balance with measurement interval: 1 mg to 300 g), and the sample was taken from the central area of the stool.

Stool samples were processed to measure fecal calprotectin levels by enzyme-linked immunosorbent assay (ELISA) using the commercially available fecal calprotectin kit produced by Euroimmun Medizinische Labordiagnostika AG (Lübeck, Germany) (recommended interval values: <50 µg/g; 50–200 µg/g and >200 µg/g for fecal calprotectin) which is intended for the detection of fecal calprotectin molecules in a sample by a sandwich type of the ELISA method (i.e., a solid phase coated with specific antigen—antibody from the analyzed sample—labeled antibody) with ADALTIS Analyzer—GEN—4 (Adaltis S.r.l, Guidonia Montecelio, Italy) and Victor X4 (PerkinElmer, Shelton, CT, USA) according to the kits’ manufacturer’s instructions. The commercially available kit used to measure fecal calprotectin level contains a labeled antibody (conjugate), which is an animal immunoglobulin fraction conjugated with horseradish peroxidase. Peroxidase activity is determined in the test by a substrate containing TMB. Positivity is indicated when the blue color appears; after the stopping solution has been added, the blue changes to yellow. The yellow color intensity is measured by a photometer at 450 nm, and it is proportional to the concentration of specific substances in the sample. Each sample was measured twice to eliminate possible pre- or intra-analytic laboratory error.

### 2.3. Used Reference Ranges

Although the laboratory analyses were performed using a commercial kit that provides its own reference values (Euroimmun Medizinische Labordiagnostika AG kit, having recommended interval values: <50 µg/g; 50–200 µg/g and >200 µg/g for fecal calprotectin), for statistical analysis we chose to apply the reference ranges that are commonly used across clinical laboratories. This decision was based on two main considerations. One is the fact that reference values provided by a specific manufacturer may vary depending on methodology, reagents, or platform. In contrast, largely accepted reference ranges, standardized and widely recognized, allow for direct comparison of our findings with data generated in most clinical practice settings, assuring inter-laboratory comparability. The other aim of this study is not the technical validation of a detection kit but rather the clinical interpretation of the results. Therefore, using broadly recognized reference intervals ensures that the findings can be meaningfully translated into real-world medical practice, where clinicians rely on standardized values.

The ranges <50 µg/g, 50–200 µg/g, and >200 µg/g used in our study are frequently used internationally for the interpretation of fecal calprotectin, being aligned with the recommendations of ECCO, NICE, and some ELISA test manufacturers (PhiCal, BÜHLMANN, IDS, etc.) in this way, reflecting both the need for alignment with routine clinical practice and the objective of ensuring comparability and external relevance of the study results [18,19,20].

### 2.4. Statistical Analyses

After inclusion–exclusion criteria, we obtained a group of 60 representative cases, 30 cases for each group (PD and IBD). Biological data, especially those related to inflammatory markers, often have an asymmetric (positively skewed) distribution, in which extreme values are not unusual, but reflect a natural variability of the disease. Thus, the use of statistical methods that automatically eliminate extreme values without clinical justification could distort the analysis; therefore, all values obtained in patients who met the inclusion criteria of the study were included in the statistical analysis. Descriptive statistics (mean, range, and percentage) were calculated for continuous variables, including age and fecal calprotectin levels. Fecal calprotectin levels were reported as means and standard deviations (SD). The Shapiro–Wilk test, used to assess data normality, revealed that fecal calprotectin values were not normally distributed, W (60) = 0.709, *p* < 0.001. Consequently, nonparametric statistical methods were further applied. Correlation analyses were conducted using Spearman’s rank correlation coefficient in IBM SPSS Statistics for Windows (Version 23.0), based on values from a specific time point (the moment patients agreed to participate in the study). Statistical significance was set at *p*-values < 0.05.

## 3. Results

### 3.1. Socio-Demographic Characterization of Patients

After applying the established inclusion and exclusion criteria, 60 patients were admitted to the study, equally distributed between the two groups (30 patients in the PD cohort and 30 patients in the IBD cohort). The demographic and paraclinical characteristics of the groups are presented in Table 1.

The mean age of patients in the PD group was 64.6 years (SD 16.54), which was 7 years older than the mean age of patients in the IBD group (57.57 years, SD 14.52). The difference in mean age between the PD and IBD patient groups aligns with established knowledge regarding the typical age of onset for these conditions. PD is a neurodegenerative disorder characterized by an inflammatory component of nervous tissue at various levels, with the incidence increasing with advancing age. In contrast, IBD is a multifactorial digestive disease marked by inflammation and specific lesions, which can manifest at younger ages.

The youngest patient diagnosed in the PD group was 23 years old, female, with a fecal calprotectin level of 597.6 µg/g, no signs of intestinal inflammation suggestive of IBD, and originating from an urban environment. Among the oldest patients in the PD group, there was a female patient aged 87 years, from rural areas, with fecal calprotectin levels over 200 µg/g (1345 µg/g) and no intestinal inflammatory changes observed endoscopically. In the IBD group, the youngest patient was a 25-year-old female from an urban environment, presenting with endoscopic lesions characteristic of ulcerative colitis (UC), moderate inflammation, and a fecal calprotectin level of 227.4 µg/g. The oldest patient in the IBD group was an 81-year-old male from an urban environment, with endoscopic findings of Crohn’s disease (CD), deep inflammatory lesions, and a fecal calprotectin level of 2971.4 µg/g. As observed, the minimum and maximum ages in the two patient cohorts are relatively similar. The standard deviations for the two groups (PD group, SD 16.54; IBD group, SD 14.52) did not reveal notable differences, reflecting the heterogeneity in patient ages across both pathologies. Interestingly, although the diagnostic age for PD is generally recognized as being over 60 years, younger patients were identified in the study cohort. Conversely, in the IBD cohort, which can present at any age, the presence of older patients is noteworthy. This may be partially attributed to the phenomenon of “inflammaging,” described in the literature as a progressive inflammatory process that develops with advancing age [14,21]. By dividing patients into age intervals corresponding to recognized stages of aging [22,23], we observe patterns characteristic of the onset of PD and IBD as described in the literature. In the PD group, the majority of patients were over 65 years old (18 patients, representing 60% of the PD cohort), followed by the 26–64 age range (11 patients, 36.67%), with only one patient (3.33%) under 25 years old. Conversely, in the IBD group, the majority of patients were in the 26–64 age range (20 patients, 66.67%), followed by those over 65 years old (9 patients, 30%), and one patient (3.33%) under 25 years old.

Gender distribution in the two groups was nearly equal between males and females. In the IBD group, there was an equal representation of genders (15 female and 15 male patients, 50% each), whereas in the PD group, there was a predominance of female patients (18 patients, representing 60% of the PD cohort) (Table 1).

A notable characteristic of the cohorts is the prevalence of patients from urban environments. In both groups, 76.67% (23 patients) of those enrolled in the study originated from urban areas, while only 23.33% (7 patients) came from rural areas (Table 1). For patients with PD, this trend may be explained by the association of symptoms with advanced age and the cultural norms of the studied population. In the case of IBD patients, dietary habits linked to urban living may represent a factor contributing to the higher proportion of urban patients in the study.

Endoscopic findings were considered an exclusion criterion for the PD patient group. Patients with endoscopic changes pathognomonic for IBD were excluded from the study. A total of 30 patients were included in the study, of whom only one presented with gastritis, while the remaining 29 showed no significant inflammatory changes. Among these, 9 patients had minor inflammatory changes in the intestinal mucosa without features indicative of IBD. The patient with gastritis had a fecal calprotectin level below 50 μg/g. Most patients with minor inflammatory lesions displayed elevated fecal calprotectin levels. Patients undergoing treatments known to influence fecal calprotectin values (e.g., non-steroidal anti-inflammatory drugs or PPI) were excluded from the study to ensure that elevated fecal calprotectin levels in PD patients could not be attributed to such factors. Additionally, given that literature reports suggest a genetic link between PD and the development of IBD, it is possible that PD patients with minimal, nonspecific intestinal inflammatory lesions and elevated fecal calprotectin levels may later develop a digestive disease [24].

In the IBD group, endoscopic findings were used both to screen patients and to classify them into specific forms of IBD. The cohort included 18 patients with CD and 12 patients with UC, aiming for a balanced distribution between the two pathologies.

### 3.2. Consideration of Fecal Calprotectin Levels in the Studied Groups

#### 3.2.1. Parkinson’s Disease Cohort

In the PD patient group, the mean fecal calprotectin level was 354.10 μg/g (SD 404.75). When stratified by age groups based on recognized aging stages, the mean fecal calprotectin levels varied across the intervals. The highest mean value was observed in the <25 years age group (597.6 μg/g), followed by the >65 years group (mean 378.06 μg/g, SD 359.29). The 26–64 years group had the lowest mean fecal calprotectin level, though it was very close to the value in the >65 years group (mean 292.75 μg/g, SD 494.50).

When age intervals are defined based on the most probable ages of PD onset [25], the mean fecal calprotectin levels for the 40–60 and >60 years intervals are reversed. In the 40–60 years age group, the mean fecal calprotectin level is 389.40 μg/g (SD 556.84), while in the >60 years group, the mean is 329.05 μg/g (SD 353.41). For the <39 years interval, the mean value remains identical to that of the <25 years group (597.6 μg/g), as only one patient was identified in this age range.

This reversal in mean values between the two older age groups is due to the reclassification of several patients into different intervals when the age ranges were redefined. Specifically, patients with fecal calprotectin levels below 50 μg/g transitioned to a different group, leading to a decrease in the mean value for that interval. A notable observation, resulting from reversed values with different definitions for age groups, is that in the studied cohort, symptomatic PD patients aged 60–65 years exhibit low fecal calprotectin levels.

**Figure 1:**
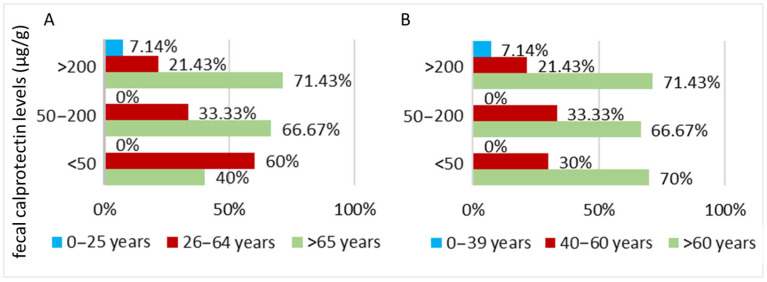
Distribution of patients with Parkinson’s disease (PD) by fecal calprotectin levels and age intervals, highlighting differences in patterns depending on the chosen classification method. (**A**) Stratified according to aging stages and (**B**) stratified according to the average age of PD onset.

Most patients with elevated fecal calprotectin levels >200 μg/g are aged over 60 years. Regardless of how the age groups are structured, these patients account for 71.43% (10 patients) of all individuals with fecal calprotectin levels above 200 μg/g, and 33.33% of the total PD cohort (Figure 1).

Analysis of fecal calprotectin levels in PD patients included in the study shows that, irrespective of the age group classification method, fecal calprotectin levels tend to increase with advancing age (Figure 1).

Analyzing the entire PD cohort, 33.33% (10 patients) had fecal calprotectin levels below 50 μg/g, 20% (6 patients) between 50 and 200 μg/g, and 46.67% (14 patients) above 200 μg/g. When the data is analyzed using age intervals based on the typical onset of PD (commonly after 60 years, with moderate probability between 40 and 60 years), this distribution remains consistent.

**Figure 2:**
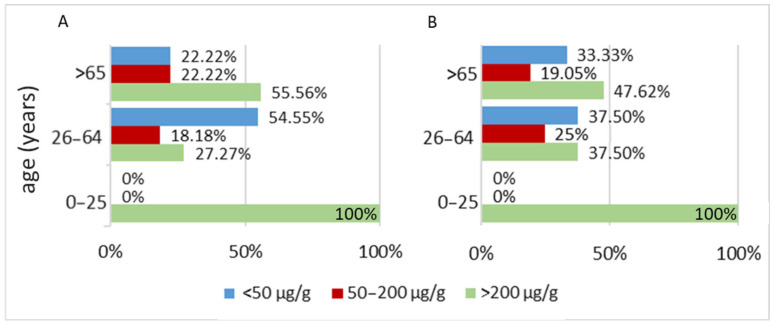
Distribution of patients with Parkinson’s disease (PD) by age intervals and fecal calprotectin levels, highlighting differences in percentages depending on the chosen stratification method. (**A**) Stratified according to aging stages and (**B**) stratified according to the average age of PD onset.

In contrast, when age groups are defined according to the stages of aging, most patients in the 26–64 years interval (6 out of 11, representing 54.55%) had fecal calprotectin levels below 50 μg/g. In the >65 years age group, most patients (10 out of 18, representing 55.56%) had fecal calprotectin levels above 200 μg/g (Figure 2).

Regardless of the age intervals used for patient stratification, in the studied cohort, the majority of patients exhibited no inflammatory signs at the level of the intestinal mucosa (12 out of 18 patients over the age of 65, and 15 out of 21 patients over the age of 60, respectively). This predominance of patients without intestinal inflammatory lesions is also maintained among younger patients. This trend is evident from the initial analysis of the studied group, where a higher prevalence of patients without intestinal inflammatory lesions can be observed (Table 1).

**Figure 3:**
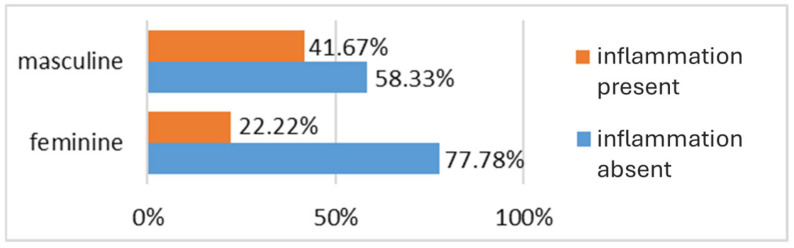
Distribution of patients with intestinal inflammatory lesions by gender.

When stratifying the patients by gender, a higher prevalence of cases without intestinal inflammation is noted in the female group (77.78% of female patients had no intestinal inflammation, compared to only 22.2% who presented with inflammation). Another notable observation is the relatively close percentage between patients without inflammation (58.33%) and those with inflammation (41.67%) as detected endoscopically in the male group. Nevertheless, the proportion of patients without inflammation remains higher even in the male group (Figure 3).

**Figure 4:**
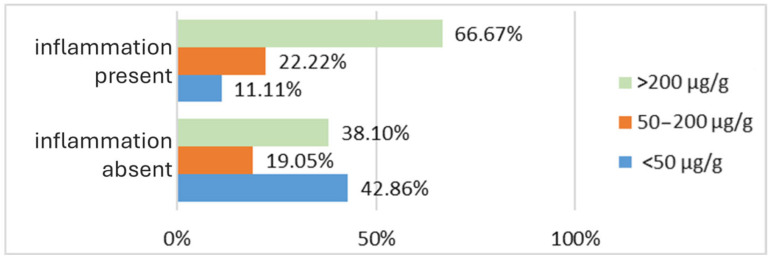
Percentage of patients with changes in fecal calprotectin values based on the presence of intestinal inflammation.

Analyzing the relationship between gastrointestinal inflammation and fecal calprotectin levels in the PD patient group, a correlation between the two variables can be observed. The majority of patients did not exhibit detectable digestive inflammatory lesions (70% of the PD cohort). Among these patients without intestinal inflammation, most had fecal calprotectin levels below 50 μg/g (42.86%). However, a considerable proportion of patients without detectable intestinal lesions also had fecal calprotectin levels exceeding 200 μg/g (38.10%), while the smallest proportion of patients without inflammatory lesions was found in the group with fecal calprotectin levels between 50 and 200 μg/g (19.05%). Interestingly, while the distribution of patients without intestinal inflammation across fecal calprotectin value groups does not inversely correlate with fecal calprotectin levels as expected, a clear trend emerges in patients with endoscopically detectable intestinal inflammation. In this group, the number of patients increases proportionally with higher fecal calprotectin values. Patients with fecal calprotectin levels below 50 μg/g represent only 11.11% of those with inflammation; those with levels between 50 and 200 μg/g account for 22.22%, and the majority (66.67%) of patients with intestinal inflammation have fecal calprotectin values exceeding 200 μg/g (Figure 4). These findings suggest the hypothesis that fecal calprotectin levels correlate more strongly with the presence of inflammation than with its absence in patients with PD.

#### 3.2.2. Inflammatory Bowel Disease Cohort

In the IBD patient group, the mean fecal calprotectin level was 537.70 μg/g (SD 795.30). When stratified by age intervals, higher mean fecal calprotectin levels were observed in patients over 60 years old (mean fecal calprotectin 601.33 μg/g, SD 877.17) compared to those aged 40–60 years (216.66 μg/g, SD 411.56). When stratified according to the aging stages, a higher fecal calprotectin value persisted in the age group over 65 years (639.81 μg/g, SD 1003.84), though the difference was attenuated compared to the mean value in the 26–64 years group (501.77 μg/g, SD 729.86). In both cases, the large standard deviations highlight the heterogeneity of fecal calprotectin values among IBD patients, regardless of age and stratification method. This heterogeneity reflects variations in disease activity and treatment response, as patients may have been in either an active disease phase or remission at the time of study inclusion.

The mean fecal calprotectin values align with the distribution of patients when stratified either by aging stages or the most probable diagnostic age range for PD. When patients were grouped by aging stages, the majority (20 patients, representing 66.67%) with IBD were in the 26–64 years age range. However, when grouped by the age ranges most associated with PD onset, the distribution shifted, with nearly equal proportions of patients in the 40–60 years age group (11 patients, 36.67%) and those over 60 years (15 patients, 50%), with a larger number of patients falling into the over-60 category.

Across the entire IBD group, most patients (13 patients, representing 43.33%) had fecal calprotectin values exceeding 200 μg/g. The patients with fecal calprotectin values under 50 μg/g and between 50–200 μg/g were almost evenly distributed (8 patients, 26.67% in the <50 μg/g group and 9 patients, 30.00% in the 50–200 μg/g group).

**Figure 5:**
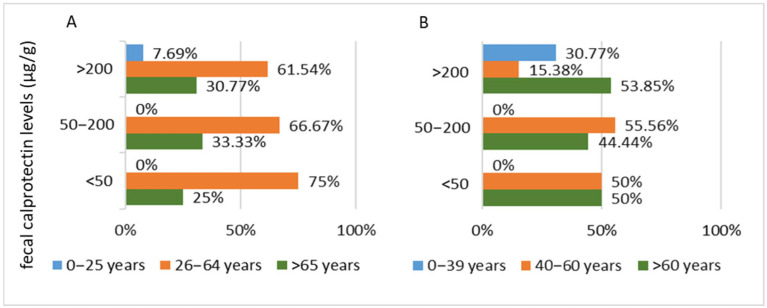
Distribution of patients in the Inflammatory bowel disease group based on defined fecal calprotectin levels. (**A**) Stratified by stages of aging and (**B**) stratified by the average age of Parkinson’s disease onset.

Within each fecal calprotectin value range, stratifying patients by aging stages revealed a predominance of patients in the 26–64 years age group (75%—6 patients in the <50 μg/g group, 66.67%—6 patients in the 50–200 μg/g group, and 61.54%—8 patients in the >200 μg/g group). However, when patients were stratified by the likely diagnostic age range for PD, the age distribution changed. For the 50–200 μg/g fecal calprotectin group, the proportions remained similar to those seen in the aging stages stratification (55.56%—5 patients in this group). In contrast, for the >200 μg/g fecal calprotectin group, the proportions favored patients over 60 years old (53.85%—7 patients in this group), reflecting the reclassification of several patients (6 patients aged 60–65 years) into the older age group (Figure 5). This pattern mirrors the distribution observed in the PD cohort (Figure 1).

**Figure 6:**
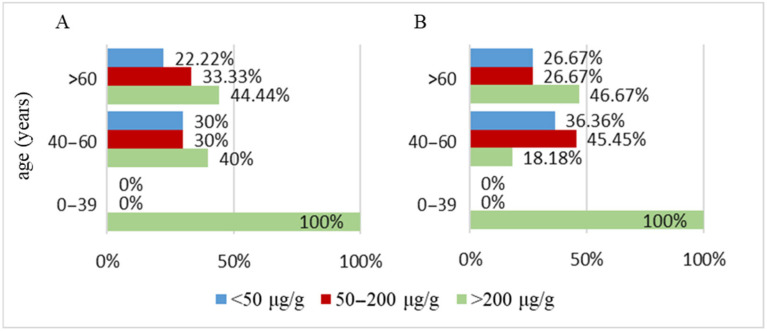
Distribution of patients with Inflammatory bowel disease by age intervals and fecal calprotectin levels, highlighting differences in percentages depending on the chosen stratification method. (**A**) Patients stratified by the age of senescence evolution, and (**B**) patients stratified by the age of Parkinson’s disease onset.

When analyzing age intervals, patients with fecal calprotectin levels exceeding 200 μg/g represent the majority in both the over 65 years group (44.44%) and the over 60 years group (46.67%), depending on the age range criteria used. In the middle-aged groups (26–64 years and 40–60 years), the proportion of patients with fecal calprotectin values exceeding 200 μg/g is higher in the 26–64 years group (40% of patients, representing 20 individuals). Conversely, in the 40–60 years group, the number of patients with fecal calprotectin levels between 50–200 μg/g is greater (45.45% of patients, representing 11 individuals). For the younger patient category (defined as up to 25 years and up to 39 years, respectively), all patients had fecal calprotectin levels exceeding 200 μg/g (Figure 6).

The age-based analysis revealed an increase in the number of CD cases with advancing age among the patients included in the study, while the number of UC cases remained relatively constant. Both conditions showed an increase in the number of cases in individuals older than 26 years. However, the same analysis did not indicate a higher prevalence of a particular degree of gastrointestinal inflammation with advancing age. This observation is primarily attributed to the inclusion of both patients in active phases of the disease and those in remission, variability in treatment compliance, and the fact that IBD can present at any age.

**Figure 7:**
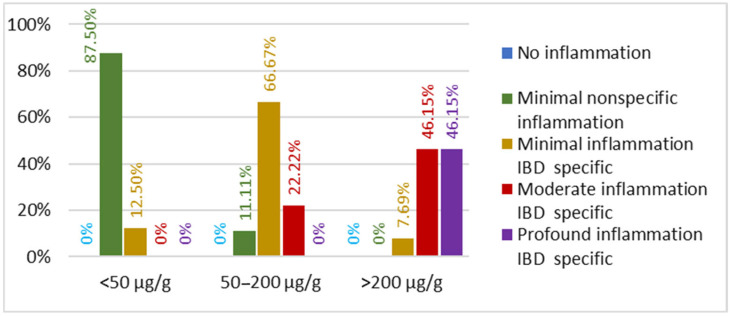
Percentage of inflammatory bowel disease patients based on fecal calprotectin values and the depth of intestinal inflammation.

Among IBD patients, different degrees of intestinal inflammation were observed depending on the disease stage. Gastrointestinal inflammation was noted to increase proportionally with fecal calprotectin levels. In the group of patients with fecal calprotectin levels below 50 µg/g, the majority presented with minimal, nonspecific inflammatory lesions (87.5% of IBD patients with fecal calprotectin < 50 µg/g), and this number decreased to zero as the degree of inflammation increased. In the group with fecal calprotectin levels between 50–200 µg/g, the majority of patients had superficial, specific inflammatory lesions (66.67% of patients). In contrast, in the group with fecal calprotectin levels exceeding 200 µg/g, patients with moderate and deep lesions were equally distributed, with each category representing 46.15% of cases. In this group, patients with superficial inflammatory lesions accounted for only 7.69% of cases (Figure 7).

These findings clearly demonstrate the relationship between fecal calprotectin levels and the degree of gastrointestinal inflammation, as reflected by the specificity and depth of the lesions.

Spearman rank-order correlation was conducted in order to evaluate the relationships between age and disease subtype, and fecal calprotectin level, and between fecal calprotectin levels and endoscopic inflammatory lesions, and endoscopic aspects of inflammation.

The analysis yielded a moderate positive correlation between fecal calprotectin levels and age in patients with PD, with a correlation coefficient of *r_s_* = 0.393 and *p* = 0.032 (Table 2). The positive correlation coefficient indicates that as age increases, fecal calprotectin levels tend to rise in this patient group. These results suggest a meaningful, though moderate, association between age and fecal calprotectin levels in PD patients, highlighting a potential link between aging and inflammatory processes.

The relationship between age and endoscopic diagnosis (UC or CD) in patients with IBD is illustrated through a strongly positive correlation, with a correlation coefficient of *r_s_* = 0.370 and *p* = 0.044 (Table 2).

The analysis revealed a positive, yet non-significant correlation between fecal calprotectin levels and the observed endoscopic inflammatory lesions in patients with PD (*r*_*s*_ = 0.282, *p*= 0.132). This suggests a weak positive association between fecal calprotectin levels and inflammation signs in the sampled patients, but with no statistical significance (Table 2).

We identified a strong positive correlation between fecal calprotectin levels and endoscopic aspects of inflammation in patients with IBD. The results showed a correlation coefficient of *r*_s_ = 0.917 and *p* < 0.001 (Table 2). The strong positive correlation indicates that higher fecal calprotectin levels are strongly associated with more severe endoscopic modifications in IBD patients.

## 4. Discussion

The aim of the research was to compare the values of fecal calprotectin between patients with IBD and those with PD, two pathologies known for involvement of the gut–brain axis. Thus, the focus was on the differences between the two pathological conditions regarding fecal calprotectin values.

A study published in 2021, conducted on data from 204 territories/counties among 21 geographic regions, highlighted that in 2019, East Asia had the highest incidence of PD (311.86 × 10^3^), while Oceania had the lowest incidence (0.87 × 10^3^) [23]. The incidence of PD has shown substantial regional variation, with increases ranging from 27.51% in Eastern Europe to 296.11% in high-income North America. In 2019, the age-standardized incidence rate (ASIR) ranged from 7.70 per 100,000 in Eastern Sub-Saharan Africa to 25.14 per 100,000 in high-income North America. Upward trends in ASIR were observed in 18 regions, with the most pronounced increase in high-income North America (estimated annual percentage change, EAPC = 2.74). A downward trend was noted only in Oceania (EAPC = −0.21). When stratified by sex, male patients consistently showed higher incidence rates and a greater increase in ASIR (EAPC = 0.80) compared with females. Across age groups, incidence was highest in individuals over 65 years, while the greatest relative increase was reported in those older than 80 years (221.67%) [26]. A Colombian study reported a 62.13% increase in prevalence when comparing individuals aged 40 years or older with those aged 50 years or older. Similarly, each additional decade of age from 50 to 80 and over was associated with prevalence increases of 83.65%, 80.95%, and 35.10%, respectively. Between 40 and 89 years, males exhibited significantly higher prevalence rates than females [27]. In the United States, a 2022 study supported by the Parkinson’s Foundation estimated that nearly 90,000 individuals are diagnosed with PD each year, reflecting a 50% increase compared to the earlier estimate of 60,000 annual cases. At all ages, the incidence of PD is consistently higher in men than in women [28]. In the United Kingdom, PD is rare among individuals under 40 years of age, with a prevalence of only 1–2 cases per 100,000 in the 20–29 age group and 4–5 cases per 100,000 in those aged 30–39. Prevalence rises sharply with advancing age, reaching 1696 per 100,000 among individuals aged 80–84 years, equivalent to approximately 1.7% of this age group. Notably, prevalence rates nearly double every five years between ages 50 and 69 in both men and women [29]. In our study, contrary to global observations regarding the prevalence of PD, the majority of patients were female (18 females versus 12 males) (Table 1). When analyzing the number of cases by age, our study aligns with previous data showing an increase in the number of patients with advancing age, with 60% of patients being over the age of 65. Among these, three patients were over 80 years old, consistent with earlier findings on the prevalence of PD. Given the concordance between our data and international statistics regarding the age distribution of PD patients, the observed gender distribution difference, where females predominate instead of males, opposite to what other statistical studies reported, may stem from the inclusion and exclusion criteria used in this study. These criteria included recent (less than 6 months prior to the study) or concurrent gastrointestinal or systemic conditions, antibiotic treatment, or the use of other drugs or supplements that may interfere with test results (e.g., steroidal or non-steroidal anti-inflammatory drugs, PPI) within the past 3 months.

Previous studies have shown that fecal calprotectin is augmented in PD patients, reflecting gut inflammation and injury to the gut barrier [14]. Gut inflammation, even at moderate yet chronic levels, may be involved in various disease processes affecting the central nervous system via the gut–brain axis and inflammaging [30,31]. Supporting the systemic relevance of gut inflammation, fecal calprotectin moderately increases to levels between 50–100 μg/g in patients with PD [14,32]. Comparing the findings of this study with the report by Sebastian Heinzel and colleagues [33], it is observed that fecal calprotectin levels in our analyzed PD patients exceed, in most cases, the values reported by Heinzel. In their report, fecal calprotectin levels were generally mildly elevated, ranging between 50–100 μg/g, whereas in our study, the most frequently observed values exceeded 200 μg/g. Notably, the PD patients in our study did not present significant endoscopic signs of gastrointestinal inflammation. Of the 30 patients enrolled, 21 showed no signs of digestive inflammation, and the 9 patients with intestinal inflammation exhibited only minimal, nonspecific inflammatory lesions. Despite this, fecal calprotectin levels were elevated in the majority of patients, with 20 patients having levels above 50 μg/g, while only 10 patients had levels below this threshold. These findings suggest the existence of factors contributing to the elevation of fecal calprotectin levels beyond gastrointestinal inflammation, such as dysbiosis or age-related inflammation (commonly referred to as “inflammaging”).

The study conducted in 2018 by A. Schwiertz and colleagues also demonstrated a high value of fecal calprotectin in patients with PD [34]. The research included 36 PD patients and 28 controls and demonstrated a higher value of fecal calprotectin (87.1 μg/g) in the tested patients. In their report, fecal calprotectin levels were generally mildly elevated with values of 87.1 μg/g and a SD of 116.9, whereas in our study, the mean value was 366.25 μg/g with a SD of 415.14, exceeding the values obtained in previous studies [14,28,30]. This may be due to the inclusion–exclusion criteria used, demographic and cultural characteristics of each group, form or duration of PD, but in all cases, the fecal calprotectin levels are above the normal value interval.

Fecal calprotectin showed no association with (prodromal) neurodegenerative diseases according to Heinzel, but a case–control study on 35 PD patients and 20 healthy controls observed that fecal calprotectin increased in 43% of PD patients compared to controls and was not correlated with duration of PD [14]. This might be due to differences between patient groups: prodromal symptoms versus established PD symptoms, even though the second study concluded that calprotectin did not correlate with the duration of the disease. Another study also showed that fecal calprotectin is increased in PD patients (n = 34) compared to healthy controls (n = 28), but does not correlate with PD severity [34]. A case–control study including 22 PD patients and 16 healthy controls illustrated that serum calprotectin was higher in PD patients compared to healthy controls; also, serum calprotectin was correlated with fecal calprotectin in PD patients [4]. These elevated serum calprotectin levels support the hypothesis that the increased fecal calprotectin values observed in PD patients may be driven by factors other than gastrointestinal inflammation.

Our study identified an increase in absolute fecal calprotectin values with advancing age, suggesting a potential age-related contribution to these elevations. A moderate positive correlation was identified between fecal calprotectin levels and age in patients with PD.

In a relatively large case–control study with 71 patients with PD and 38 patients with multiple system atrophy (MSA), compared to 60 matched controls without neurological disorders, fecal calprotectin was increased in both patients with PD and MSA compared to controls. However, fecal calprotectin was significantly correlated with patients’ age over 61 years, and immune blood biomarkers, but not with clinical presentation [35].

This type of correlation between elevated fecal calprotectin levels and the age of over 61 years in PD patients can also be observed in the current study, as age-based stratification of patients leads to percentage changes in the patients with fecal calprotectin levels exceeding 200 μg/g, according to the selected age intervals (over 60 years or over 64 years) (Figure 3).

To the information provided by previous studies, the current study adds observations regarding the increase in fecal calprotectin with age in patients with PD and the correlation of this increase, especially with exceeding the age of 61. These, taken together, indicate that fecal calprotectin is increased in PD and could be a potential indicator for the presence of gut-barrier dysfunction or other mechanisms.

Age in isolation appeared to have a significant cross-sectional effect on fecal calprotectin concentration in a study on 77 PD patients and 113 controls [21]. In this study, any aging effect on fecal calprotectin concentration is confounded by iatrogenic inflammaging, namely PPI exposure [21]. In our study, PD patients with PPI exposure were excluded, but the effect of high fecal calprotectin with age remained visible. In the cited study, fecal calprotectin level was 34 μg/g in PD patients, and the group also included patients treated with PPI or NSAI, compared to our study, where the calprotectin mean level in the PD cohort was 366.25 μg/g. The above study focused on fecal metabolite deficit, gut inflammation clinical phenotype, including colonic transit time and diet in PD patients with a cutoff point of >50 μg/g for the presence of histological activity and 188 μg/g for ‘clinically significant inflammation’ according to the authors of the study, who cited the work of JhaAK and QinXY. Calprotectin expression may be regulated by stress in addition to inflammatory factors [35]. Thus, high plasma and fecal calprotectin levels in PD may be due to psychological stress and hypothalamic–pituitary–adrenal axis dysfunction, and associated GIT disorders.

In our study, gastrointestinal inflammatory lesions (other than those that can be categorized as IBD) are present in 9 patients out of the 30 in the group with PD. We could not identify a significant correlation between fecal calprotectin levels and the observed endoscopic modifications of inflammation in patients with PD.

Gastrointestinal tract symptoms are linked to the hypothalamic–pituitary–adrenal axis in healthy subjects, and in 2016 Ibrahimagic et al. showed that dysfunction of the hypothalamic–pituitary–adrenal axis is associated with advanced PD due to psychological stress [36,37]. Most PD patients have gastrointestinal disturbances such as constipation and delayed gastric emptying that precede motor symptoms by decades due to disturbances of the enteric nervous system (ENS) of the GIT, and fecal calprotectin is mainly interrelated with gut-immune dysfunction in the old-age group, signifying an age-dependent effect of fecal calprotectin [8,38,39].

In a study on inflammatory cytokines in PD patients (19 PD patients and 14 age-matched healthy controls), correlation analyses showed that pro-inflammatory cytokines were upregulated in the ascending and descending colon of PD patients, suggesting that only a subset of PD patients had an “enteric pro-inflammatory profile” [40,41] which would explain the lack of endoscopic images suggestive of inflammatory lesions in some of the patients studied in our group. Considering this aspect, fecal calprotectin increase in PD may have other mechanisms besides those highlighted by studies from 2019 and 2022 according to which gut dysbiosis together with environmental toxins determine oxidative stress, intestinal mucosal inflammation and accumulation of α-Syn in the ENS leading to injury of gut barrier and leaky gut which enhance transfer of local inflammatory mediators causing systemic inflammation and neuroinflammation so that gut dysbiosis is correlated with increasing of fecal calprotectin [42,43,44,45].

In Rolli’s review, Derkinderen et al. (2019) support the idea that gastrointestinal inflammation is more likely to occur in patients with a short disease duration [40]. Unfortunately, our study cannot differentiate between patients with a short period of disease evolution and those with a long period because the determination of fecal calprotectin was made at the first presentation, and the history of the disease was taken from the patient’s statements.

In a literature review [40], the idea that PD and IBD are genetically related is supported and argued, PD being in fact a form of IBD, specifically CD, with neurological manifestations due to systemic inflammation.

Considering this option, compared to the group of PD patients, we analyzed a group of patients with IBD (both UC and CD) who did not present symptoms suggestive of PD, and we tried to find correlations between the same types of parameters. The analyzed IBD cohort has age characteristics similar to the PD cohort. We identified a strongly positive correlation between age and endoscopic diagnosis (UC or CD) in patients with IBD. Inflammatory bowel disease is recognized as a group of diseases that can occur at a young age and in older individuals as well. In our patient cohort, aging appears to be associated with changes characteristic of a specific IBD subtype, namely an increase in the number of CD cases with advancing age, while the number of UC cases remains relatively constant. This observation may be attributed to other group-specific characteristics, such as local customs, health education, and preventive health practices within the tested population. Further studies could help elucidate the clinical implications of this association.

Although there seems to be a correlation between age and the predominance of a specific IBD subtype, patient age does not correlate with the degree of inflammation in IBD patients in our study. Patients with the same disease subtype may present at different stages of disease progression, exhibiting varying degrees of gastrointestinal inflammation. These aspects are influenced by individual patient characteristics and treatment compliance. Additionally, fecal calprotectin levels did not show a significant correlation with patient age or with the endoscopic features pathognomonic for UC or CD. However, in the PD cohort, fecal calprotectin levels were found to correlate with age, with values increasing as patients aged. This finding is also supported by other studies [21,46]. The fact that fecal calprotectin values do not correlate with the diagnosis of UC or CD confirms the results of other studies that claim that fecal calprotectin values cannot differentiate between the two IBD forms [13,47,48,49,50]. The current study included both patients with active disease and progressive lesions, as well as patients in remission. Regarding the degree of inflammation identified endoscopically, fecal calprotectin showed a positive correlation with increasing levels of inflammation and lesion severity, regardless of the IBD entity (UC or CD). This correlation between fecal calprotectin levels and the degree of gastrointestinal inflammation was also observed, albeit as a weak positive correlation, in the cohort of PD patients. The correlation coefficient indicates a very strong positive association, meaning that higher fecal calprotectin levels are strongly associated with more severe endoscopic modifications in IBD patients. These findings underscore the strong relationship between fecal calprotectin levels and endoscopic disease activity, supporting fecal calprotectin’s role as a reliable and statistically significant biomarker for assessing mucosal inflammation in IBD.

Over time, various studies have reported various cutoff values, useful in differentiating IBD patients from those with other diseases that can increase fecal calprotectin values [47,51,52,53,54]. This could influence the level of correlation between fecal calprotectin and other parameters in PD patients. In the current study, the cutoff values recommended by the kit manufacturer were used (below 50 μg/g for normal, 50–200 μg/g for mild gastrointestinal inflammation, and above 200 μg/g for significant gastrointestinal inflammation). Based on our observations, a connection between PD and IBD, as suggested by Rolli et al. [40], cannot be supported, since only clinical data were analyzed, and paraclinical data related to systemic inflammation and genetic factors could not be evaluated. Indeed, genetic studies shed a different light, as the shared genetic background implicated in the etiopathogenesis of the two diseases could change diagnostic and therapeutic approaches, including how fecal calprotectin is regarded as a common marker for both conditions.

By comparing the mean fecal calprotectin values between PD and IBD patients and analyzing their correlation with the degree of inflammation identified endoscopically, we observed similar fecal calprotectin values between the two groups (354.10 μg/g in the PD group and 537.70 μg/g in the IBD group). However, IBD patients exhibited a higher mean fecal calprotectin value, which corresponds to more pronounced endoscopic inflammatory lesions.

According to previously cited studies, PD patients have elevated fecal calprotectin values but rarely exceed 200 μg/g. In contrast, in our study, most PD patients had fecal calprotectin values exceeding 200 μg/g. In the study of Jia Wei Hor and co. [46], fecal calprotectin level had a median of 95.6 μg/g in PD patients, which is below the median value obtained in our study (366.25 μg/g) for PD patients. According to the cited study, 18.3% of PD patients (71 patients) had highly abnormal fecal calprotectin levels (≥250 μg/g) [46]. This cutoff value is higher than the one used in our study. Had the same cutoff been used, their reported percentage would probably have been similar to the 46.6% observed in our study (46.6% of PD patients with calprotectin level over 200 μg/g). Given the low levels of gastrointestinal inflammation in PD patients and the weak positive correlation between fecal calprotectin levels and intestinal inflammation, it can be inferred that fecal calprotectin levels in PD patients increase due to factors other than gastrointestinal inflammation. This suggests that elevated fecal calprotectin levels in PD patients may be linked to mechanisms involving the hypothalamic–pituitary–adrenal (HPA) axis and other stress-related factors, alongside intestinal permeability alterations, gut microbiota dysbiosis, and the presence of inflammatory lesions.

Unfortunately, the study is limited by the small sample size (30 patients), and the inclusion–exclusion criteria, which differ from those used in previous studies, may lead to discrepancies or false consistencies between the current findings and those of other studies. Previous studies have used comparisons of PD patient groups with healthy patient groups. We preferred to compare patients from groups with pathologies recognized as related to the brain–gut axis, and we used IBD, knowing that fecal calprotectin is a marker used in the diagnosis of the disease. This comparison between different groups may limit the results of the study, also in terms of the influence of inflammaging on fecal calprotectin values.

Although fecal calprotectin levels may fluctuate over time, influenced by factors such as diet, subclinical infections or variations in intestinal activity, in this study, a single determination per patient was chosen, even though this limits the results of the study. The choice was motivated by the desire to reflect the inflammatory intestinal state at a standardized, clinically relevant time point, and to ensure the practical feasibility of the study, given the available resources. This protocol is also consistent with common clinical practice, where therapeutic decisions are frequently based on a single measurement, especially when this is supported by the clinical context. Patients included in the study in the two groups were assessed using internationally accepted scales to confirm the fulfillment of the criteria for belonging to a certain pathology. Also, previous studies are largely based on a single determination per patient.

Larger studies with clear, standardized inclusion and exclusion criteria are needed to eliminate possible variations caused by cohort-specific characteristics.

## 5. Conclusions

Fecal calprotectin values were higher in PD compared to those reported in previous studies, frequently exceeding 200 μg/g (mean being 366.25 μg/g), possibly due to the demographic characteristics of the cohort and the inclusion/exclusion criteria.

The gender distribution of PD patients in this study (mostly women) differs from international trends (where men predominate).

Fecal calprotectin levels increase with age in patients with PD, suggesting the influence of aging (inflammaging) on this marker.

No clear link has been identified between age and the degree of inflammation in IBD, although a trend towards an increase in Crohn’s cases in older ages has been observed.

Fecal calprotectin levels in PD patients are slightly lower but are comparable to those observed in patients with inflammatory bowel disease (UC, CD).

Fecal calprotectin is elevated in patients with Parkinson’s disease (PD), even in the absence of clear endoscopic signs of gastrointestinal inflammation.

Compared with IBD, fecal calprotectin in PD is less influenced by active intestinal inflammation, suggesting different mechanisms for the elevation of this biomarker.

Elevated fecal calprotectin levels in PD may be influenced by predisposing factors such as dysbiosis, psychological stress, or hypothalamic–pituitary–adrenal (HPA) axis dysfunction.

This gastrointestinal-specific marker could potentially be useful for diagnosing PD patients after excluding digestive disorders that elevate fecal calprotectin levels.

Study limitations include a small sample size, lack of clustering by disease duration, and a single determination of fecal calprotectin.

## Figures and Tables

**Table 1:** Social and paraclinical characteristics of studied cases (PD—Parkinson’s Disease; IBD—Inflammatory bowel disease).

Parameter	Social Data/ Paraclinical Data	PD Group	IBD Group
		N/Value (SD)	
Provenience area	Rural	7	7
	Urban	23	23
Age—mean		64.6 (16.54)	57.57 (14.52)
Age	Minimum	23	25
	Maximum	87	81
Gender	Feminine	18	15
	Masculine	12	15
Fecal Calprotectin (µg/g)—mean value		366.25 (415.14)	537.70 (795.30)
Fecal Calprotectin (µg/g)—median value		184.62	167.85
Fecal calprotectin (µg/g)—value	Minimum	23.33	0.1
	Maximum	1345	2971.4
Fecal Calprotectin— number of cases	<50 µg/g	10	8
	50–200 µg/g	6	9
	>200 µg/g	14	13
Endoscopic diagnostic	Without IBD-specific inflammatory changes	29	0
	UC inflammatory aspect	0	12
	CD inflammatory aspect	0	18
	Polyps or diverticulosis	0	0
	Gastritis and ulcers	1	0
Inflammation level	No inflammation	21	0
	Minimal nonspecific inflammation	9	8
	Minimal inflammation IBD-specific	0	8
	Moderate inflammation IBD-specific	0	8
	Profound inflammation IBD-specific	0	6

**Table 2:** Spearman’s Rank-Order correlation coefficients and significance levels in the Inflammatory Bowel disease (IBD, n = 30) and Parkinson’s Disease (PD, n = 30) cohorts.

Cohort	Variable 1	Variable 2	r	*p*
IBD patients	age	disease subtype	0.370	0.044
	fecal calprotectin	endoscopic aspects of inflammation	0.917	0.000
PD patients	age	fecal calprotectin	0.393	0.032
	fecal calprotectin	endoscopic inflammatory lesions	0.282	0.132

## Data Availability

Data is contained within the article.

## Abbreviations

The following abbreviations are used in this manuscript:

IBD	Inflammatory bowel disease
PD	Parkinson’s disease
GIT	Gastrointestinal tract
IBS	Irritable bowel syndrome
UC	Ulcerative colitis
CD	Crohn’s disease
PPI	Proton pump inhibitors
ASIR	Age-standardized incidence rate
EAPC	Estimated annual percentage change
HPA	Hypothalamic–pituitary–adrenal axis
